# Four Cultural Narratives for Managing Social-ecological Complexity in Public Natural Resource Management

**DOI:** 10.1007/s00267-020-01320-6

**Published:** 2020-07-07

**Authors:** Nick A. Kirsop-Taylor, Adam P. Hejnowicz, Karen Scott

**Affiliations:** 1 Politics Department, University of Exeter, Penryn, Cornwall, TR10 9FE UK; 2 Department of Biology, University of York, Wentworth Way, YO10 5DD UK

**Keywords:** Public agency, Water–Energy–Food nexus, Organisation, Environment, Culture, Social-ecological complexity

## Abstract

Public Natural Resource Management (NRM) agencies operate in complex social-ecological domains. These complexities proliferate unpredictably therefore investigating and supporting the ability of public agencies to respond effectively is increasingly important. However, understanding how public NRM agencies innovate and restructure to negotiate the range of particular complexities they face is an under researched field. One particular conceptualisation of the social-ecological complexities facing NRM agencies that is of growing influence is the Water–Energy–Food (WEF) nexus. Yet, as a tool to frame and understand those complexities it has limitations. Specifically, it overlooks how NRMs respond institutionally to these social-ecological complexities in the context of economic and organisational challenges—thus creating a gap in the literature. Current debates in public administration can be brought to bear here. Using an organisational cultures approach, this paper reports on a case study with a national NRM agency to investigate how they are attempting to transform institutionally to respond to complexity in challenging times. The research involved 12 elite interviews with senior leaders from Natural Resources Wales, (NRW) and investigated how cultural narratives are being explicitly and implicitly constructed and mobilised to this end. The research identified four distinct and sequential cultural narratives: collaboration, communication, trust, and empowerment where each narrative supported the delivery of different dimensions of NRW’s social-ecological complexity mandate. Counter to the current managerialist approaches in public administration, these results suggest that the empowerment of expert bureaucrats is important in responding effectively to complexity.

## Introduction

The significant majority of contemporary public organisations operate in increasingly complex policy domains (Cairney et al. 2019). They must negotiate issues arising from an array of social, economic, and ecological systems and the interactions between them (Capra and Luisi 2016). Complex systems such as these are highly diverse and dynamic by nature and characterised by properties such as non-linearity, multiscalarity, feedbacks, tipping points, self-organisation, emergence, path dependency, adaptation, and uncertainty (Mobus and Kalton 2014). Consequently, because they are open to continual change and interact with other systems in unanticipated ways, complex systems display a high degree of unpredictability in their responses to different drivers of change, which makes the task of managing and governing them particularly demanding (Young 2017).

Public natural resource management (NRM) agencies, in particular, face a difficult situation in managing multiple complexities (Kennedy and Quigley 1998; Belcher 2001; Koontz and Bodine 2008). First, like other organisations, they have to address organisational and operational complexities such as human resource and strategic development issues (Stacey 2015) and executive accountabilities (Thomann et al. 2017; Gravey et al. 2018; Schoenefeld and Jordan 2019). Second, like other public policy delivery agencies, they have to tackle the complexities of policy implementation and evaluation (Cairney et al. 2019) whilst under the increasing pressure of political and economic challenges and public expectations (Van Wart 2013; Taylor et al. 2019; National Audit Office 2018). Third, they have an additional layer of complexity to manage, in the form of the local and global sustainability challenges such as those posed by climate change, biodiversity loss, and land-use change (e.g., Vince 2014; Steffen et al. 2018) that involve the governance of complex social-ecological systems (Young 2017). In other words, interrelated environmental systems (e.g., marine, freshwater, and terrestrial ecosystems) and social systems (e.g., fisheries, agriculture, and forestry) (Cortner et al. 1998; Belcher 2001; Rammel et al. 2007; Biggs et al. 2015; Young 2017).

These complexities proliferate unpredictably (at the time of writing we are in the middle of the COVID-19 pandemic), therefore investigating and supporting the ability of public agencies to respond effectively is increasingly important (Eppel and Rhodes 2018). However, understanding how public NRM agencies innovate and restructure to negotiate the range of particular complexities they face is an under researched field. One particular conceptualisation of the social-ecological complexities facing NRMs that is of growing influence is the Water–Energy–Food (WEF) nexus. Yet, as a tool to frame and understand those complexities it has limitations. Specifically, it overlooks how NRMs respond institutionally to these complexities in the context of economic and organisational challenges—thus creating a gap in the literature. Current debates in public administration can be brought to bear here. Using an organisational cultures approach, this paper reports on a case study with a national NRM agency to investigate how they are attempting to transform institutionally to respond to complexity in challenging times. The research involved senior leaders from the Welsh national natural resource agency—Natural Resources Wales (NRW), and focusses on how narratives are being explicitly and implicitly constructed to create a better organisational culture for addressing complexity.

## Responding to Complexity

### Socio-ecological Complexity Frameworks

Understanding how organisations in general develop, operate, respond, and behave falls within the realm of organisational studies and public administration (Stacey 2015; Mullins 2016). Traditionally, public administration scholarship has focused on organisational and operational complexity and how increased challenges in service delivery often test the boundaries of political trust and bureaucratic autonomy and empowerment (Peters 2010: 29–72; Thomann et al. 2017). Some scholars work from the perspective that rising complexity is best managed through an increasingly professional bureaucracy who can make autonomous expert situational decisions (Randolph 1995; Jamil et al. 2016; Kim and Fernandez 2015). Others argue from more managerial approaches that stricter accountabilities will endow bureaucrats with the tools necessary to meet complex situations (e.g., audit cultures) (Halligan 2007; Bovens et al. 2014; Schillermans, van Twist 2016). These differing perspectives reflect a wider debate about the optimal modality for exercising control and ensuring accountability in a bureaucracy (Peters 2010: 263–302).

More recently, there has been a broadening of scope beyond these traditional perspectives, including an increased focus on how public NRMs govern in relation to the complex social-ecological systems under their remit (Barton et al. 2010; Cilliers et al. 2013; Scott et al. 2015). A challenge that demands significant organisational and epistemic change from NRM agencies that are often sectorally organised, have difficulty integrating social and natural science research, and remain at the whim of political economies and ideologies (Leck et al. 2015). The ecosystem approach, arising from the Convention on Biological Diversity (Jenkins et al. 2015), provided an early attempt to design a holistic, non-sectoral, and decentralised framework for integrated NRM based on a suite of fundamental principles (CBD 1998; Waylen et al. 2015). Indeed, as we outline later, the ecosystem approach was the foundation NRW adopted as an organisational framing to navigate social-ecological complexity (Kirsop-Taylor and Hejnowicz 2020). In relation to natural resource use, scarcity, and management, the WEF nexus provides a more recent conceptual framing of social-ecological complexity that has garnered widespread policy traction (e.g., Ringler et al. 2013; Scott et al. 2015). In many respects, the WEF nexus represents a contemporary social-ecological problematic that previously saw efforts at reconciliation through the ecosystem approach (Leck et al. 2015; Bhaduri et al. 2015; Bizikova et al. 2013).

The WEF emphasises the complex interconnections between related biophysical systems (i.e., water, energy, and food), economic sectors, and policy domains as they affect human wellbeing and public welfare (Scott et al. 2015). In that regard, the WEF provides an approach to think systematically (so-called ‘nexus thinking’) about the interdependencies underlying the functioning of social-ecological systems as well as a means to adopt multi-disciplinary systems perspectives (Ringler et al. 2013; Leck et al. 2015; Albrecht et al. 2018). Whilst the utilisation of the WEF as a particular framing of contemporary social-ecological challenges and a form of enquiry is not without criticism (Wiegleb and Bruns 2018; Simpson and Jewitt 2019) it resonates strongly with national policy-actors (Leck et al. 2015; Kirsop-Taylor and Hejnowicz 2020).

Hence, there is increasing emphasis on understanding complex social-ecological challenges through a WEF lens (Dodds and Bartram 2016). And, in particular, addressing the challenges posed by interconnected and interdependent systems through a governance imperative (Weitz et al. 2017; Pahl-Wostl 2019). However, whilst the WEF is particularly useful as a means of examining biophysical and cross-sectoral linkages within a system, in relation to the issue of governance, the WEF is generally more concerned with understanding the external role and actions of actors, such as NRM agencies, involved in governing social-ecological systems. As such, it offers limited insights into how public NRM agencies should internally reconcile emerging knowledges of and accountabilities for social-ecological complexity alongside the economic, political, and operational challenges of maintaining funding, capacity, and capabilities. In addition, the focus on system perspectives for organisational change management, which often goes hand in hand with the WEF approach, has been criticised for its rationalist and reductionist approach (Tsoukas and Hatch 2001; Mowles et al. 2008; Simpson 2012). The tendency to frame organisations as a set of structures and agents where different levers can be pulled by change managers neglects the ‘day-to-day difficulties of trying to achieve things together, which is what it would mean to understand the process of organising as complex processes of relating’ (Mowles et al. 2008: 816; Simpson 2012). In these circumstances, which concern matters of internal organisational dynamics and behavioural responses, it is necessary to turn to other approaches that can be applied to examine these issues, notably ‘organisational culture’ (Peters 2010: 33–78).

### Organisational Culture

There is a long-held view that the field of public administration should be considered as a form of culture science (e.g., *Kulturwissenschaft*—see Ringeling 2017) that acknowledges the criticality of culture in shaping the public sphere. Within this perspective public organisational culture is a versatile and powerful theoretical framing for understanding how public organisations manage and reconcile multiple complexities (e.g., Parker and Bradley 2000; Parry and Proctor-Thomson 2010; Stanford 2010; Dartey-Baah et al. 2011; Lowndes and Roberts 2013). Culture exists at all levels of an organisation (Rez and Gati 2004) and in relation to all issues and operations. Therefore, it is a useful lens to examine both broadly and deeply across multiple complex and intersecting domains within organisations. Smircich (1983) reviewed the cultural turn in organisational theory and developed a five-pronged typology to categorise understandings of organisational culture. In order to bring clarity to the field she linked ontological assumptions about culture and organisations from anthropology and organisational theory respectively to create five views of organisational culture: comparative management, corporate culture, organisational cognition, organisational symbolism, unconscious processes and organisation. This typology allows researchers to interrogate their own ontological understandings of both ‘culture’ and ‘organisation’. In this paper we broadly follow an organisational cognition model where organisations are ‘systems of knowledge’ and culture is a ‘system of shared cognitions’ where both systems function in relation to ‘rules’ (Smircich 1983: 342). This resonates closely with Schein’s well-known definition of organisational culture:

‘Culture can now be defined as (a) a pattern of basic assumptions, (b) invented, discovered, or developed by a given group, (c) as it learns to cope with its problems of external adaptation and internal integration, (d) that has worked well enough to be considered valid and, therefore (e) is to be taught to new members as the (f) correct way to perceive, think, and feel in relation to those problems’. (Schein 1990, p 113)

Viewed in this manner, culture can help and encourage the building of adaptivity and learning into public organisations (Costanza et al. 2015). Equally, organisational cultures can help organisations transition away from toxic and intolerant practices (e.g., UK Police foundation 2018) towards cultures that tolerate mistakes as gateways to learning and innovation (Betts and Holden 2004; Maria 2003; Wodcka-Hyjek 2014; Olejarski et al. 2019). The embedding of cultural values has also been identified to be significant for business performance and management in the face of external threats (Mansol et al. 2014), as well as moderating internal organisational behavioural dynamics and communication (Fischer and Smith 2006; Sagiv and Schwartz 2007).

### Cultural Narratives

In recent decades there has been a steadily increasing academic and policy interest in the potential power that narratives have in shaping, informing, and constructing organisational cultures (Doolin 2003; Rowlinson et al. 2014) for improving management (Browning 1991; Rhodes and Brown 2005), especially during periods of change and complexity (Bevir and Krupicka 2007; Strandberg and Vigsø 2016). Narratives have been shown to influence policy-making (e.g., Rhodes 2002; Stevens 2011; Lowndes 2016), constitute evidence (Epstein et al. 2014), and shape policy cultures (e.g., Rhodes 2018). Organisational cultural narratives are the accounts of connected events and ideas that create a value-laden story relevant to a particular collections of individuals (Eisenberg et al. 2001; Dettori 2011; Utoft 2020). They are the stories that help to build, change, and/or sustain shared meanings in an organisation. Organisational cultural narratives can manifest as crisp linear stories (Labov 1972; Hones 1998) or as mosaics of commonly associated collection of themes, aspirations, and observations that collectively account and construct the ‘associative determinants’ of narrative (as per Fulford 1999).

Whilst certainly there is a critical counter-literature about narrative approaches to policy analysis (e.g., Jones and McBeth 2010), the weight of scholarly activity in this field is focussed on how narrative approaches can offer an important conceptual lens for understanding complexity in contemporary public agencies (Browning 1991; Lämsä and Sintonen 2006; Denning 2011; Pekar 2011; Morrell 2006; Dalhstrom 2014; Vaara et al. 2016). The proliferation of narrative approaches to organisational studies (and to a lesser degree public administration) literatures has facilitated an increasing sophistication in the methods of narrative analysis (e.g., Riessman 1993; Daiute and Lightfoot 2004). In a systematic review Sahni and Sinha (2016) acknowledge the growing use of narrative approaches in organisational analysis but find that understanding the role and significance of narratives within organisations is an under researched field. Furthermore, there are few contributions to this literature exploring narratives for culture in public NRM agencies (e.g., Dalhstrom 2014) who, as already noted, face a somewhat unique set of challenges.

We build on the view that narrative themes that influence communications, organisational relationships, visions and values, and where leaders are deeply embedded in a process of change, can be the basis for new types of organisational cultures to emerge (Simpson 2012; Stacey 2015). We therefore argue that cultural narratives should be a useful tool for constructing or re-aligning public organisational cultures towards the challenges of grappling with social-ecological complexities. As mentioned above, popular system-based approaches for change management in organisations dealing with uncertainty and complexity have limitations. Simpson (2012) argues that these approaches tend to characterise leadership as the key to success, usually framing this as a one-step removed change management ‘hero’ that can use their expertise of how systems work to guide the organisation through difficult transitions. Instead they argue for attention to ‘the evolving dynamics of relating that make an organisation what it is and how it is continuously evolving’. That is a focus on the multiple everyday interactions through language that build complex patterns of how an organisation thinks about itself, what it can achieve and how it should act. We explore this argument through the case of NRW.

## Case Study

### NRM and the Challenges of the Current UK Political Landscape

The United Kingdom (UK) offers an interesting setting for understanding complexity in public NRM agencies. Since 2016 until the time of writing the discourse regarding agency capabilities for dealing with complexity has focused on their preparedness for ‘Brexit’ i.e., the UK’s departure from the European Union (EU) (e.g., Rutter and McCrae 2016; Jessop 2017) and the requirement for new and replacement policies (see: UK Government’s Industrial Strategy and 25-Year Environment Plan, both launched in 2018). Moreover, when combined with systematic agency underfunding following 10 years of public sector austerity (National Audit Office 2018; Kirsop-Taylor et al. 2020) and uncertainties around post-Brexit ‘zombie legislation’ (Burns and Carter 2018: 25) the current situation is particularly challenging. These relatively ‘acute’ political and economic issues co-exist, and to some degree overshadow, the longer-term challenge of responding to and governing social-ecological complexity through remodelling how public NRM agencies function and behave (Kirsop-Taylor and Hejnowicz 2020).

### History and Development of NRW

**Fig. 1:**
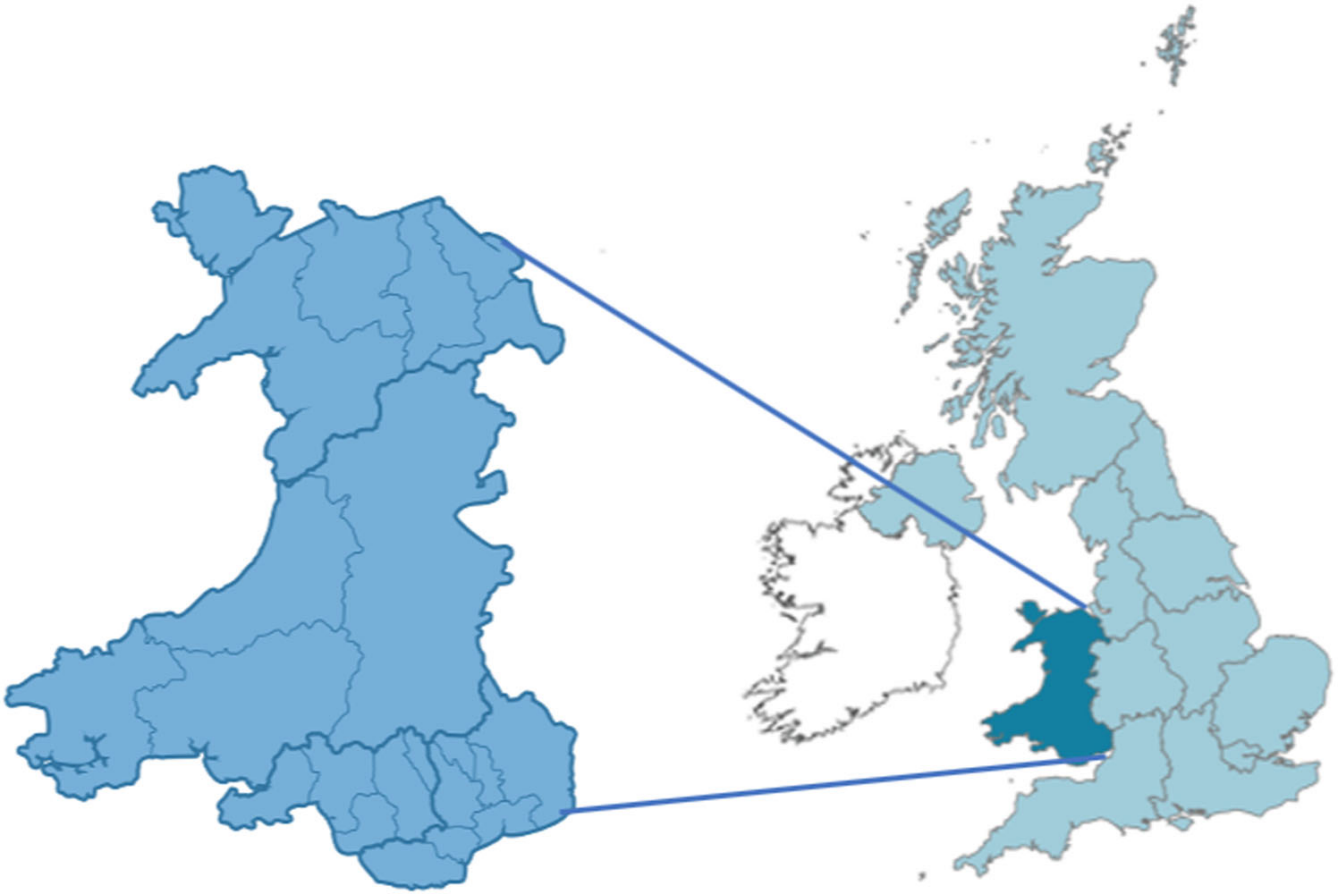
Devolved Wales. Source: Business Wales 2019

Within the UK Wales (see Fig. 1) is a (non-federal) nation that operates under a reserved model of devolved governance. This provides a degree of power for drafting legislation, managing budgets, and setting rules within certain policy areas (Trench 2015) and the fully devolved ‘environment’ policy-area. Following a widespread consultation and listening exercise the ‘Sustaining a Living Wales’ green paper (2012) articulated the challenges and vision for twenty-first century NRM agency in Wales. This led to an ambitious policy and institution-building agenda (St.Denny 2016; Moon and Evans 2017) legislated through the Wellbeing of Future Generations Act (2015) and the Environment (Wales) Act (2016).

**Fig. 2:**
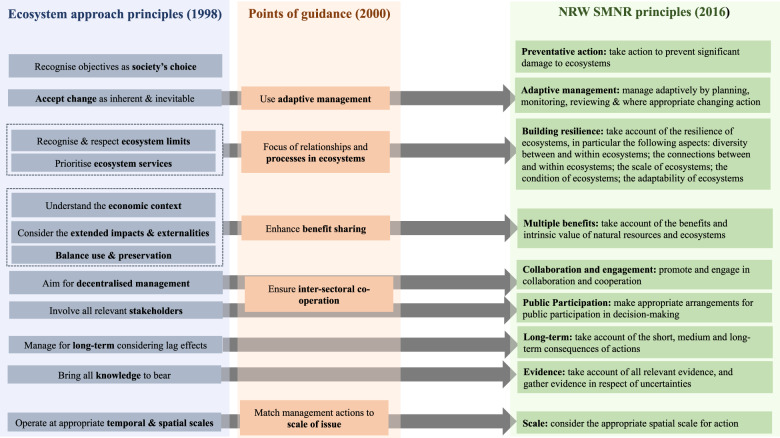
From the Convention on Biological Diversity’s ecosystem approach to NRW’s principles of SMNR

The Environment (Wales) Act (2016) built on previous ambitions^1^ by consolidating the three separate NRM agencies (the Welsh Environment Agency, the Countryside Council for Wales, and the Welsh Forestry Commission) into a unified national agency called NRW. Each of the legacy agencies brought unique and differentiated activities, processes, leadership styles, and cultures to the new agency (Waylen et al. 2015). The Environment (Wales) Act (2016) legislated for the design and adoption of a new multiple-scale framework approach for national NRM mindful of the need to be better attuned to social-ecological complexity (as expressed in the consultation). This new approach, called the ‘sustainable management of natural resources’ (SMNR), was based upon instantiating an adapted form of the Malawi Principles of the ecosystem approach from the Convention on Biological Diversity (Jenkins et al. 2015). Figure 2 highlights how NRW adapted the principles of an ecosystem approach into their SMNR programme to meet the challenges posed by managing social-ecological complexity.

The principles of the SMNR approach are activated by NRW through a mix of discretionary and legislative powers, and executive expectations for implementation. The operationalisation of these principles into the processes, architectures, and activities of NRW is an evolving process of learning, iteration, and adaptation.

### Justification for Focusing on NRW

We selected NRW as a case study for a unique combination of reasons, which together provide a useful and interesting context in which to consider the role of cultural narratives in building and facilitating organisational performance for complexity. First, they are a newly created NRM organisation with a fresh organisational mandate. Second, they are currently explicitly transforming their organisational processes and functions to increase their complexity-capacity through the SMNR approach. Third, they are at a global first-mover disadvantage in implementing the SMNR approach. Fourth, they have a level of political patronage that has provided an enabling environment to act and operate in an innovative way. Moreover, they are operating within highly fiscally constrained conditions as a result of public austerity. No UK public NRM agency has faced such a significant disparity of funding between their initial business case (2013) and current funding settlements as NRW (Reynolds and Ninnes 2017). Finally, as noted by Waylen et al. (2015), organisational legacies, such as those from the three agencies that constituted NRW, can create hurdles for integration and innovation that require significant effort to overcome (Kirsop-Taylor and Hejnowicz 2020). In NRW this has produced an environment ripe for structural, functional, and cultural innovation, as well as for contention, failure(s), and potential for learning.

Whilst the combination of justifications for case selction evidence the uniqueness of NRW as a case, the challenges it faces in terms of meeting socio-ecological complexities, change management, and building culture are near universal to all other international NRM agencies. Therefore, this case-based research may help other organisations interested in such transformational aspirations and facing similar conditions and constraints, to anticipate and understand the opportunities and barriers involved in such processes from an organisational cultures perspective.

## Method

The focus of this research was on organisational cultural narratives to create more effective responses to complexity. Similar research within large public agencies has highlighted the value of the elite interview method for understanding complex organisational change management situations (Schara and Common 2016). Hochschild (2009) notes that fewer focussed interviews with respondents chosen for their detailed knowledge of a subject are likely to yield richer data than other sampling approaches. Congruent with the normative rationale of elite case-based research methods (Leuffen 2007; Luton 2010: 26-28; Boggards 2018) this small sample of elite managers were, in all probability, the only interviewees who could offer such detailed insights into the phenomena under investigation. They were the ones tasked with, and responsible for, organisational change with a focus on changing organisational culture and the organisational messaging around that. This therefore necessitated a qualitative research design with a small *n* sample of NRW organisational elites (as per Luton 2010). The drawbacks of elite interviewing in terms of accessibility, positionality, and small *n* sample sizes (Harvey 2011) were offset by the benefits of gaining first-hand accounts that were highly detailed and nuanced.

Twelve semi-structured interviews were conducted with senior managers within NRW. The primary contact within NRW was established through an existing organisational gatekeeper, which led to opportunity and snowball sampling. The interview sample comprised members of the senior management team (*n* = 3), departmental heads (*n* = 4), team leaders (*n* = 2), and senior members of the change management team (*n* = 3). The sample included representatives from each of the three legacy agencies that comprised NRW, and from former members of Welsh Government now working in NRW. A semi-structured interview method was employed that raised specific issues whilst giving flexibility for elite-led dialogue (see Supporting Information). Empirical data were collected between March and May 2018 through a combination of Skype based interviews and telephone calls (see Supporting Information). Interviews were recorded using the *italk* application and produced 10 h of data for transcription and analysis. The data were analysed in *NVivo 11* (QSR International 2018) against a partially pre-set, but emergent and iterative node framework based on parent nodes such as ‘culture’, and child nodes such as ‘leadership’, ‘legacies’, and ‘narratives of culture’ (see Supporting Information).

## Findings

Interviewees considered that the development of an effective and appropriate culture was essential in meeting their legislative mandate to govern complex socio-ecological systems. They expressed how the development of culture in NRW was a long-term project that incorporated both existing activities, as well as aspirational elements.

### Organisational Culture and Leadership

Members of the senior team at NRW had a solid understanding of what the SMNR principles were and how they helped them address social-ecological complexities. Seven interviewees considered that whilst building the processes, forms and structures of the new joint agency was important, these would ‘only take NRW so far in its journey’ (Int. 3) and all interviewees considered ‘culture’ to be a critical element in delivering the SMNR approach.

Most interviewees expressed the view that culture change in the organisation was necessary to develop shared understandings of SMNR and how to deliver it. This was achievable through education, training, mentoring, coaching, collaboration, and communication:

‘We’ve got the training, bringing everybody up to a common understanding of what it all means … We’ll continue on that for the next couple of years as we go through the next phase of organisational design. There is a growing understanding that SMNR is the place where we could start to move away from our legacy traditions and build the new NRW traditions and culture. [Interview 5]

A majority of ten interviewees were critical of the notion that organisational culture could be created in a top-down fashion. Some of these interviewees pointed towards the annual NRW ‘People surveys’ of 2015 and 2016 as evidence of staff perceptions of the coercive nature of management during the formation stage of NRW. Nine interviewees expressed the view that the emergence of culture was a natural evolutionary process that was hard to create though imposition. However, there was some recognition that the pressing legislative mandate and political pressures for delivering the SMNR approach necessitated and legitimised top-down efforts at shaping or supporting culture, even if this entailed a degree of coercion. Interviewee Four described shaping culture ‘a nettle that we have to quickly grasp’ (Int. 4) and Interviewee Seven noted:

‘it’s hard to build a culture for an organisation which has gone through rapid change, perhaps it needs something stronger’

Three interviewees indicated that their new organisational culture was a long-term project that would only emerge through ‘a supportive environment’ as Interviewee Eight noted:

‘I think you can create the conditions to allow culture to emerge, though I think you can’t necessarily shape it through a hard process. It takes time, it’s a lot of time, but you can certainly create the conditions for emergence’.

Unsurprisingly, several interviewees (*n* = 5) suggested that this ‘supportive environment’ had to be constructed at the intersection of senior management leadership, and the values and behaviours of bureaucrats in NRW as a whole. Eight interviewees drew attention to the critical role of the agency leadership and leaders in taking responsibility for the emergence of a culture to meet complexity. Six interviewees described the characteristics of leadership in terms of being able to communicate a consistent vision for the agency (*n* = 3). Two interviewees expressed that their role was to work to create an organisational sense of shared mission and endeavour, or what Interviewee Nine called ‘a sense of us’. As Interviewee Eight said:

‘I think leadership vision is critical. The troops … are quite attached to some of their old stuff and they see it [leadership for culture] as corporate nonsense. It takes a little while before you get used to it and you realise … that it’s not corporate nonsense. I think it’s critical to support in the future of the organisation.’

### Narratives for Culture Change

Interviewees offered a range of comments about narratives for cultural change. Seven interviewees noted a number of different and intersecting narratives about addressing social-ecological complexity through SMNR. Four of these interviewees further argued that these narratives were key to building the culture that NRW needed to adopt if it was going to meet its legislative mandate:

‘We have to be better at building the stories to demonstrate the difference that it [SMNR] makes. It’s about the narrative… If we tell the stories in a more engaging way, in a more live, real way … and the real difference that the SMNR outcome will have, that is the trick that we need to be able to pull off, I think’. [Interview 5]

‘It [culture change] does take a while and you have to be really consistent in narrative. You have to essentially keep saying the same thing for a long time.’ [Interview 2]

‘It’s all about making sure people are doing the right thing. A lot of this is our own narrative. Narrative is much more important, in my experience of working around the UK … I think [this] is the way to make sure that we get this metamorphosis or accept change into doing the right things … I don’t think that the way that people using some behavioural insights work is probably the way that we will make sure the organisation is moving in the right direction.’ [Interview 12]

Interviewees generally expressed a sense that the culture of NRW would necessarily emerge from aspects of the legacy cultures that comprised NRW, and from the activities of the different parts of the organisation. Four of the interviewees argued that there were widely understood stories that constructed a sense of organisational identity and purpose attached to each of the legacy agencies, for example, Interviewee Eight noted how:

‘the story of the Countryside Council for Wales being ‘the advocates’, the Forestry Commission being ‘the problem-solvers’, and the Welsh Environment Agency being ‘the enforcers’; and that these should never meet, or could never get on was quite powerful’.

Three interviewees commented on how narratives about organisational identity that constructed the legacy agencies could now act as barriers for working together to meet social-ecological complexity in the new agency, for example, Interviewee Four who noted how:

‘I think you will always have teams with a different focus. I wouldn’t say, I wouldn’t talk about them (cultures) emerging. I think it’s more a case of how do we overcome the different cultures of the three agencies so far and some people find that easier than others depending where you were in that agency. People in environment agency don’t have any trouble understanding the Countryside Council of Wales biodiversity people.’

### Narrative Themes for Creating NRW Culture

We found that the above discussions coalesced around four interconnected narrative themes about the identity and purpose of the agency: communication, collaboration, trust, and empowerment. Whilst we review these themes separately for clarity and expediency, they need to be appreciated in terms of their integration with one another, and it is important to note how these themes are interconnected and variously interwoven through discussions of cultural change. This can be illustrated in the aspirations of Interviewee Eight below which was a fairly typical narrative for cultural change. This includes common themes of changing the structure of the organisation for greater *communication* and *collaboration*, and *empowering* employees to take risks and having *trust* they will not be punished for this:

‘This is a question that I’m really keen to explore actually is how do you evaluate how connected a person or an organisation is? … It’s about how can you really demonstrate that connectedness is which I’m convinced is an [important]part of dealing with complexity … I would like there to be less structure around teams and disciplines and more multi-workplace based teams, multi-disciplinary teams, and really keen to work horizontally across the organisation with other parts of the business to try those outcomes and also externally taking perhaps more risks to get to those outcomes.’

**Fig. 3:**
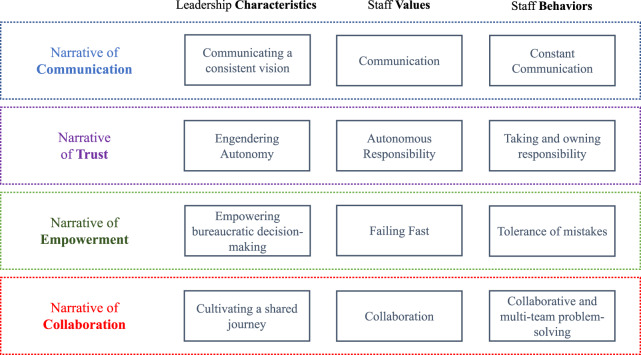
Four cultural narratives of NRW

Seven interviewees (in different ways) considered that these narrative themes might be key in forming an agency-level culture. Three other interviewees expressed how these narratives could help them deliver SMNR and meet the emerging social-ecological challenges identified. Respondents discussed various activities and responsibilities conducted by particular actors to help build and or deliver these narratives. These discussions covered what the leadership said and did, and the behaviours and values of individuals and teams in the whole organisation. There was a differentiation between those narrative themes that were already commonly adopted by the organisation, and those that the leadership aspired towards. Therefore, a narrative pattern emerged about who NRW already were culturally, and who they might yet become to meet SMNR. Figure 3 shows the four distinct, yet connected, narratives that NRW elite interviewees considered were already evident, or aspired towards. We now discuss each of those narrative themes in turn acknowledging that the narrative themes were interwoven with each other.

#### Communication

Interviewees conceptualised NRW becoming a communicative organisation in terms of how they, the leadership, would have to communicate consistently about a unified cultural vision for the organisation. They expressed a hope that this endeavour would feed into the wider evolving value system of the organisation, which could be evidenced through staff behaviours inclined towards high levels of communication in relation to problem-solving activities. Four Interviewees commented on how individual behaviours would also need to reflect a culture of rapid, open, and free-flowing interdisciplinary communications. Interviewee Nine offered a comment that captured this:

‘we should be an organisation that talks to itself at every level, with no barriers.’

This included a belief that communication would have to be framed as a story about regularity of contact and co-creation of solutions to complexity through team discourse (Int. 8). This was part of a wider conversation about how the success of this approach would be evidenced in the collaborative and multi-team behaviours team members displayed (*n* = 3). Interviewee One discussed this in terms of:

‘I know it’s not all about structures, but we are trying to design structures that would make (communication) easier, we’re looking to bring (people) together in (local area-based) teams. …. then connect upwards to another more national team to provide the overarching policies and priorities for the whole of Wales … culturally its in people’s behaviours and things like that—we’ve still got a way to go.’

#### Collaboration

Interviewees considered their role in part was to foster a sense of NRW being an intensely professional agency that had a culture of continual learning and growing individual expertise through strong joint working:

‘That is the biggest improvement that I have to achieve before I retire, that we’ve changed the culture of the organisation in such a positive way that the whole thing works as a system…We’ve got a holistic system within the organisation that enables everybody to work together to the common good, if you like.’ (Interview 6)

The interview discourse revealed how the narrative of Collaboration is already evident in the actions and messages of the NRW leadership:

‘If you look at the areas around collaboration and integration, those principles there, that requires an open culture of listening to others. It also requires business processes and system constraints that happen with the organisation to allow those things to take place… [If] You have individual targets on performance. If you develop a very individually competitive culture within an organisation, nobody’s ever going to collaborate.’ [Interview 3]

This narrative has likely already been mobilised, as seen in values and behaviours of the NRW team—evidenced in part by the findings of the 2015 and 2016 ‘People surveys’, the process of delivering the NRW State of Natural Resources Report and ‘flattening’ the structure of the organisation into place-based area teams (as shown in Kirsop-Taylor and Hejnowicz 2020).

‘The second version of that is the area statements, which we’re doing at the moment. They are bringing much more staff together which creates the numbers. You can see that culture prodding out now between the eco side of things.’ [Interview 4]

Four interviewees discussed how NRW needed to foster a staff-wide sense that collaboration was essential to solving complex social-ecological challenges. Three of these interviewees considered that collaboration had to be more than just a behaviour that could be easily witnessed by communication practices, but a deep value that shaped all decision-making and activity.

#### Trust

Interviewees considered Trust as a multi-directional dynamic that infused all levels of NRW, and was manifested in three principal areas. It was primarily considered in terms of how the leadership offered trust to the wider organisation based on their skills, experiences, and capacities to make autonomous decisions free from direct and instantaneous oversight. Others considered that the leadership characteristics would also have to reflect a sense of Trust in the wider organisation to own and take responsibility for mistakes when they occur. For two other interviewees there was a third dimension to Trust, that bureaucrats could trust the leadership to deliver a certainty of vision. The story of Trust in NRW was considered as a bidirectional and reciprocal relationship between bureaucrats and leadership—so as to loosen the overly prescribed bonds of accountability in the interests of increasing agility, responsiveness, and innovation. As Interviewee Eleven described:

‘we trust you, you trust us, and together we all act quickly, decisively, and innovate in response to complex problems.’

Trust was seen as multi-dimensional, and conceived in terms of intra-organisational *social* trust and extra-organisational *public* trust, or, as Interviewee Ten suggested: ‘we trust each other, and the public should trust us’. Interviewees considered this would make NRW more resilient and adaptive to evolving WEF problems and accountabilities, as well as more likely to be evidence-based in their decision-making. Three interviewees expressed how NRW could not meet its legislated mandate to deliver SMNR without a culture of trust that circumvented (legacy) managerialism and associated cultures of blame and risk aversion. Critically, however, they considered that a narrative of Trust needed to be partnered with, and lead to, a narrative of Empowerment.

#### Empowerment

Four interviewees described how a key characteristic of leadership in NRW should be to trust members of the team to problem-solve complex issues. For two interviewees this was predicated upon a growing sense of bureaucratic professionalism and expertise for meeting complexity through training, learning, and development. Four interviewees perceived that this had to be coupled with a responsibility to help empower agency members to go beyond traditional knowledges towards interdisciplinarity, for example Interviewee Nine who argued that:

‘we need to be helping colleagues feel comfortable with going out of their intellectual comfort zone, which let’s be honest, is mostly based on what they’ve learnt before!’

Three other interviewees also expressed how part of their role as leaders was to set a climate in which team members felt empowered to use their increased professionalism and expertise to make informed expert decisions without fear of blame. Two others argued how despite this being a difficult challenge, due to the nature of UK civil service culture, this was a key job for them as leaders if they were going to trust individuals to problem-solve complex issues.

Interviewees also described how the culture for addressing complexity would need to be actualised through the behaviours that individual members of NRW team displayed. This included the behaviours associated with increased individual autonomy (*n* = 3). Coupled to the notion of increased autonomy were comments (*n* = 3) about the fear of failure that comes with increased autonomy. Two interviewees related how they had to therefore build a narrative about an acceptance of failure, insomuch as failure is a gateway to rapid learning or what Interviewee Three described as ‘failing fast’.

Interviewees also discussed the underlying values that would support these behaviours and characteristics of leadership. Two other interviewees expressed comments on how re-framing failure as a positive outcome needed to be deeply embedded in values that individuals held. For example, Interviewee Six argued that if this was not the case ‘it wouldn’t work, people need to *know* that getting it wrong is OK sometimes’. The depth of internalised values was expressed by Interviewee Four when describing how they wanted leaders to have a genuine ‘tolerance of mistakes’, or Interviewee Nine (one of three interviewees) who argued that the flipside of tolerance was responsibility, and that ‘we need everyone to own their mistakes’. Two interviewees noted that this would have to be linked to evidence of learning and improvement to assuage executive and public expectations for accountability; though the natural conflict this might cause with the narrative for *Trust* was not discussed.

## Discussion

The development of a certain ‘culture’ was an explicit theme in the aspirations of NRW leadership for the future. The interviewees’ conceptions of culture and their desire to explicitly change/develop/work with certain cultures resonated well with Schein’s definition as set out in the introduction. Congruent with Parker and Bradley (2000), Parry and Proctor-Thomson (2010), Stanford (2010), and others (see: Schein 1990; Dartey-Baah et al. 2011; Lowndes and Roberts 2013) we found that interviewees considered culture a critical component in building a successful public NRM organisation. Interviewees considered that whilst leadership in public NRM agencies might play an important role in creating support structures for the development of a particular culture (as per Parker and Bradley 2000), the delivery and emergence of culture had to be a collaborative endeavour across all layers of the organisation (Rez and Gati 2004; Mowles et al. 2008). As noted in the Introduction, the general paucity of literature on organisational cultural narratives in public NRM agencies (and especially those seeking to tackle social-ecological challenges) made the nature of Findings, and this Discussion, in many ways exploratory in nature. Narratives for cultural change were expressed in the form of sequential and associative structure in the discussed themes, aspirations, and expectations of the discourse, as per Fulford’s (1999) account of organisational narratives.

### Organisational Cultural Narratives for Sociological Complexity

Of course, the question remains how will/can these narratives be practically employed to help NRW develop as an organisation in a form attuned to handle social-ecological complexity? In Table 1, we outline how the four distinctive cultural narratives we identified can help facilitate and instantiate specific SMNR principles (illustrated in Fig. 2), thus providing a pathway to enable NRW to meet its statutory mandate and the challenges of governing complex social-ecological resource systems.

**Table 1:** Narratives facilitating SMNR

Narrative	SMNR principles
Communication	Adaptivity, participation, multiple benefits
Trust	Resilience, adaptivity, evidence
Empowerment	All
Collaboration	Collaboration, participation, multiple benefits, long-term

Our findings chime with Pekar (2011) in that interviewees considered that building characteristics, values, and behaviours that would make NRW a communicative organisation were perhaps one of easier endeavours. The value of communication in social-ecological management activities has similarly been well-documented (e.g., Johnson and Karlberg 2017) as has the value of communicative public organisations (e.g., Canel and Luoma-aho 2018). Interviewees expressed aspirations for NRW being a ‘communicative organisation by nature’ (Int. 1) that moved beyond pure public relations-style communications to inculcating a narrative of ongoing open and honest dialogue with citizens and colleagues.

The narrative of Collaboration (both within NRW and between NRW and other agencies) was found to be the most important cultural narrative element for meeting aspects of social-ecological complexity, a finding supported by the wider literature (e.g., Ringler et al. 2013; Leck et al. 2015; Tanaguchi et al. 2017). Mobilising the narrative of a collaborative organisation has the potential to help NRW meet their SMNR mandate of being participatory, recognising the multiple benefits from natural resource decision-making, and managing for the long-term. Though, of course, simply mobilising a narrative in a superficial way would not necessarily be the sole and automatic determinant of it happening. A theme which emerged from the findings was a desire to have these narrative themes emerge from and embedded in internalised values rather than a more superficial, top-down, abstract change management strategy. As such this chimes well with Stacey’s work and arguments about narrative and change processes in organisations (Stacey 2015; Mowles et al. 2008).

The discussions of narrative themes around trust and empowerment were interesting where it engaged with the aforementioned debate in public administration theory about the optimal modalities for ensuring accountability (Peters 2010: 263–302). There is a longstanding tension in how public agencies and bureaucrats are held accountable between the degree of autonomy they enjoy to make professional decisions free from oversight; and the degree of control that their controlling authority holds them under (see: Romzek and Dubnick 1987). Excessive bureaucratic autonomy can lead to the emergence of unaccountable elite cadre, but in contrast limited bureaucratic autonomy can lead to inflexibility, risk aversion, and calcification (Leyden and Link 1993). Betts and Holden (2004) and Kittle (2017) have noted how limiting autonomy can precipitate bureaucratic risk aversion which can, in due course, stymie risk-taking as an opportunity for organisational learning. There is long and well-developed public administration literature highlighting the criticality of trust within complex public organisations (Hindmoor 2002; Chen et al. 2013; Brown 2018). In this case, elites considered that NRW are mobilising this narrative so that it might become an organisation that exhibits trust between all members of the organisation. This trust will act as a facilitator for the connected cultural narrative of empowerment.

Interviewees suggested that a cultural story of acceptance towards reasonable failure should be promoted where it supported NRW adopting the wider outcome of becoming a learning organisation (as per Maria 2003; Betts and Holden 2004; Jamil et al. 2016; Olejarski et al. 2019). This was not described by interviewees as a ‘no blame culture’ (see: Boviard and Quirk 2013; Wise 2018) such as that seen in the UK Police Foundation (2018). Instead the intention expressed by interviewees was for a form of cultural story that NRW would tell about itself in which bureaucrats were empowered to make complex decisions based on their training and knowledge and blame for failures could be apportioned insomuch as failure was reframed as a learning exercise.

Interviewees did not consider the narrative of Empowerment as being associated with meeting any specific aspect of SMNR (and more broadly social-ecological complexity). Rather, Empowerment was considered a critical determinant for individual bureaucrats deploying their skills and experiences to make complex decisions without fear of over-management or excessive blame for mistakes. If NRW could conceptualise itself as an organisation that trusts and empowers its staff to make decisions, take responsibility, fail fast, and that tolerates mistakes, then it might be able to meet current and future social-ecological sustainability challenges. This builds upon Kirsop-Taylor (2018) who suggested that meeting today’s complex sustainability challenges might require a reconceptualisation of public managerialism (e.g., notions of public and executive accountability, and empowerment of bureaucrats) in NRM agencies, and that the failure to do this might result in agencies becoming unresponsive and increasingly ineffective in meeting their respective mandates.

To the majority of interviewees there was a sequential and layered nature to these narratives, with Trust and Empowerment being built upon a foundation of Collaboration and Communication. This sequential conceptualisation meant that some interviewees already considered that NRW were exhibiting the narratives of Collaboration and Communication, that a narrative of Trust was starting to emerge, and that potentially in the future this might lead to NRW telling itself the story of its Empowerment. This means that NRW is more likely to meet the SMNR principles of Adaptivity, Collaboration, Participation, Long-term, and Multiple benefits in the short term; whilst the principles of Adaptivity, Resilience, and Evidence might take longer (as the narratives of Trust and Empowerment are mobilised). Thus, what was discerned in this research offers a snapshot of a process of narrative evolution within NRW towards a more conscious effort to create a culture to meet contemporary social-ecological challenges.

## Conclusion

In this paper we have offered an original empirical contribution to extant theorisation about the use of narratives in public NRM organisations. It advances current understandings about how public NRM agencies can adapt reflexively to new and emerging complexity challenges through culture and narrative. The findings of our research suggest that the narratives of Communication, Collaboration, Trust and Empowerment might be utilised to mobilise the cultural changes needed to meet complex configurations of social-ecological expectations within challenging political and economic contexts. Collaboration was highlighted to be the central cultural narrative for encouraging and developing a coherent organisational level of a common and recognisable mutual culture. Our results also discerned a social-ecological driven dynamic tension between the narratives of Empowerment and Trust, and prevailing norms of bureaucratic accountability. This tension suggests a loosening of the bonds of formal accountabilities in favour of greater informal accountabilities driven by the need for bureaucrats to be empowered to manage and solve social-ecological complexity at the street level. This is an emancipatory aspiration that would make professional bureaucrats more accountable to informal mandates of expertise and professionalism as opposed to strict formal accountability.

Public NRM agencies internationally are facing these same challenges and the findings here from one leading-edge organisation have highlighted one approach and its emergent impacts. The counter to this might suggest that the increasing social-ecological complexity pressures might be met through other innovations such as natural capital approaches or tighter and developed managerialism. This might precipitate a conflict of ideas about how best to respond to meeting social-ecological complexity—through the re-empowerment of bureaucrats or doubling-down on managerialism. To recapitulate, there is increasing emphasis on formulating NRM social-ecological complexities and challenges through a WEF lens, which has more recently focused on the importance of advancing the consideration of the governance foundations of these interconnected and interdependent systems. Consideration of social-ecological complexities through a WEF lens can be useful to articulate, in particular, the physical and cross-sectoral connections within these systems, and for examining how actors operate within these systems from an external perspective. However, it offers very limited insights into how actors such as public NRM agencies should, at an institutional level and from an internal organisational dynamic, reconcile emerging knowledges, and accountabilities for complexity with the economic, political, and operational challenges of maintaining funding, capacities, and capabilities. Systems-based approaches which have gained popularity with public agencies to help them deal with complexity and change management often fail to deal with the everyday realities and complexities of organisational culture. We stress the importance of further research as vital to inform these debates and to understand how NRM agencies can respond effectively to the increasing expectations on them in the challenging political and economic context of the 21st century. Ultimately, the findings offer important insights for public administrators attempting to address social-ecological complexity within their agency; and for Public Administration scholars at the intersection of narrative, organisation, and culture.

## Supplementary information

Supplementary Information

## Footnotes

1 Especially the ‘Natural Resources Body for Wales Establishment Order’ (2012)
