# Supplementary material for: Four Cultural Narratives for Managing Social-ecological Complexity in Public Natural Resource Management
Source: Environ Manage. 2020 Jul 7;66(3):419–34. doi: 10.1007/s00267-020-01320-6 (PMC7338285; doi:10.1007/s00267-020-01320-6)
Supplement: Supplementary file 1 — Supplementary Information [file 267_2020_1320_MOESM1_ESM.docx]

Four cultural narratives for managing complexity in public natural resource management.

Supporting information

**One**. Semi-structured interview questions

| Question order: | Question: |
| --- | --- |
| Question One | What do you understand an ecosystem approach or SMNR to mean? |
| Question Two | Why does SMNR help you address Water-Energy-Food orientated complexity challenges? |
| Question Three | What do you understand complexity to mean in the context of the governance and management of natural resources? |
| Question Four | How are NRW operationalising SMNR? |
| Question Five | In what way does SMNR help to address the governance and management of natural resources across different disciplines, and or scales? |
| Question Six | Can you describe the ‘journey’ that NRW are on in terms of instituting the processes, forms, and thinking of SMNR; where are NRW on this journey, what is next, and where is the ‘destination’? |
| Question Seven | In what way have the different organisational legacy cultures that different colleagues brought to NRW been transformed into new and uniquely NRW style of leadership, processes, and culture? |
| Question Eight | How would you describe the culture within NRW? |
| Question Nine | How does this culture account for socio-ecological complexity? |
| Question Ten | How are NRW evaluating their internal operationalisation of SMNR? |
| Question Eleven | How does the organisational culture(s) affect how NRW evaluates itself? |
| Question Twelve | What are the most significant future challenges facing NRW, its culture, and delivering its complexity mandate? |

**Two.** Interview schedule

| Identifier | Position | Method | Date | Duration (mins) | |
| --- | --- | --- | --- | --- | --- |
| One | Senior Advisor | Skype | 02/04/18 | | 0.55 |
| Two | Senior Economist | Skype | 05/04/2018 | | 0.47 |
| Three | Principal Advisor | Skype | 06/04/2018 | | 0.39 |
| Four | Head of continuous improvement | Telephone | 06/04/2018 | | 1.12 |
| Five | Team Leader | Skype | 11/04/2018 | | 0.58 |
| Six | Senior Advisor | Skype | 12/04/2018 | | 0.43 |
| Seven | Programme Manager | Skype | 16/04/2018 | | 0.56 |
| Eight | Senior Research Officer | Skype | 16/04/2018 | | 0.41 |
| Nine | Head of Corporate Planning and Governance | Skype | 23/04/2018 | | 1.06 |
| Ten | Portfolio Director | Skype | 25/04/2018 | | 1.01 |
| Eleven | Senior Adviser | Telephone | 25/04/2018 | | 0.55 |
| Twelve | Senior Advisor | Skype | 13/05/2018 | | 1.30 |

**Three**. Data analysis nodes

| Parent node | Child nodes |
| --- | --- |
| SMNR/EA for managing nexus complexity | Legal/statutory imperative |
|  | Originality & Effectiveness |
|  | Advantages/disadvantages of ESA/SMNR |
|  | ‘First-mover’ advantage/challenges |
|  | Nexus complexity challenges  Journey towards SMNR  Water-Energy-Food nexus |
| About NRW | Legacy(ies) |
|  | Challenges and opportunities of ‘new’ agency |
|  | Processes, artefacts, architectures |
|  | Clean sheet-ism |
| Culture in NRW | Why culture? |
|  | Can culture be influenced/affected? |
|  | Witnessing culture |
|  | Culture and bureaucrats |
|  | Leadership |
|  | Culture and process |
|  | Culture and structure |
|  | Legacy ‘sticking points’ |
|  | Narratives of culture |
